# Detection of Heart Sounds in Children with and without Pulmonary Arterial Hypertension―Daubechies Wavelets Approach

**DOI:** 10.1371/journal.pone.0143146

**Published:** 2015-12-02

**Authors:** Mohamed Elgendi, Shine Kumar, Long Guo, Jennifer Rutledge, James Y. Coe, Roger Zemp, Dale Schuurmans, Ian Adatia

**Affiliations:** 1 Department of Mathematics and Computing Science, University of Alberta, Edmonton, Canada; 2 Pediatric Pulmonary Hypertension Service and Cardiac Critical Care, Stollery children’s Hospital, Mazankowski Heart Institute, University of Alberta, Edmonton, Canada; 3 Department of Pediatrics, Stollery Children’s Hospital, University of Alberta, Edmonton, Canada; 4 Department of Biomedical Electrical and Computer Engineering, University of Alberta, Edmonton, Canada; Shenzhen institutes of advanced technology, CHINA

## Abstract

**Background:**

Automatic detection of the 1^st^ (S1) and 2^nd^ (S2) heart sounds is difficult, and existing algorithms are imprecise. We sought to develop a wavelet-based algorithm for the detection of S1 and S2 in children with and without pulmonary arterial hypertension (PAH).

**Method:**

Heart sounds were recorded at the second left intercostal space and the cardiac apex with a digital stethoscope simultaneously with pulmonary arterial pressure (PAP). We developed a Daubechies wavelet algorithm for the automatic detection of S1 and S2 using the wavelet coefficient ‘*D*_6_’ based on power spectral analysis. We compared our algorithm with four other Daubechies wavelet-based algorithms published by Liang, Kumar, Wang, and Zhong. We annotated S1 and S2 from an audiovisual examination of the phonocardiographic tracing by two trained cardiologists and the observation that in all subjects systole was shorter than diastole.

**Results:**

We studied 22 subjects (9 males and 13 females, median age 6 years, range 0.25–19). Eleven subjects had a mean PAP < 25 mmHg. Eleven subjects had PAH with a mean PAP ≥ 25 mmHg. All subjects had a pulmonary artery wedge pressure ≤ 15 mmHg. The sensitivity (SE) and positive predictivity (+P) of our algorithm were 70% and 68%, respectively. In comparison, the SE and +P of Liang were 59% and 42%, Kumar 19% and 12%, Wang 50% and 45%, and Zhong 43% and 53%, respectively. Our algorithm demonstrated robustness and outperformed the other methods up to a signal-to-noise ratio (SNR) of 10 dB. For all algorithms, detection errors arose from low-amplitude peaks, fast heart rates, low signal-to-noise ratio, and fixed thresholds.

**Conclusion:**

Our algorithm for the detection of S1 and S2 improves the performance of existing Daubechies-based algorithms and justifies the use of the wavelet coefficient ‘*D*_6_’ through power spectral analysis. Also, the robustness despite ambient noise may improve real world clinical performance.

## Introduction

New digital stethoscopes provide opportunities to improve clinical cardiovascular diagnosis. In particular, the transformation of acoustic data coupled with machine learning algorithms offer the potential to develop automatic systems for auscultation-based diagnosis [1–4]. Pulmonary arterial hypertension (PAH) is difficult to diagnose clinically, and many patients complain of symptoms for 2 years before a diagnosis is made [5]. Definitive diagnosis of PAH often requires an invasive cardiac catheterization which is not widely available and carries risk especially for children [6]. Therefore, there would be considerable merit to a non invasive screening tool to aid in the diagnosis and appropriate referral of children with suspected to PAH to an expert center. Pulmonary arterial hypertension has characteristic heart sounds that make it an ideal disease to diagnose noninvasively by auscultation using machine learning algorithms [2]. Prior to developing an automated diagnostic algorithm, it is important to accurately and reproducibly detect the first and second heart sounds (S1 and S2), which audibly separate systole from diastole. The accurate automated detection of S1 and S2 heart sounds remains a difficult problem in heart sound analysis [7, 8].

Previous attempts to detect S1 and S2 by signal processing have adopted two main approaches, which are differentiated by whether or not they need a simultaneous electrocardiographic signal (ECG). The ECG is used as a reference, and the timing between the QRS complex and the T wave in the cardiac cycle is exploited to identify S1 and S2 [3]. However, in clinical practice, noisy ECG signals may make automated ECG wave detection difficult. Therefore, many researchers have tried to identify S1 and S2 without using a reference ECG signal [2, 9]. Several signal processing techniques have been attempted, such as artificial neural networks [10], decision trees [11], envelograms [12], quantified spectrograms [13], self-organizing maps using Mel frequency cepstrum coefficients [14], and pseudo affine Wigner–Ville distribution [15, 16]. The most common technique used in heart sound analysis is discrete wavelet transform [17]; in particular, Daubechies wavelet, as researchers with its reported its superiority for detecting S1 and S2 [3, 8].

As the choice of wavelet type is still debatable [18], we sought to focus only on investigating the four well-known Daubechies wavelet-based algorithms that claimed better performance in detecting heart sounds. During our analysis, we developed a novel event-related algorithm for the automated detection of S1 and S2 without using the ECG as a reference. In addition, we sought to determine the best algorithm for detecting S1 and S2 in children by quantitatively comparing the wavelet-based algorithms.

## Materials and Methods

### Ethics Statement

The Research Ethics Board of the University of Alberta approved the study. All subjects over 18 years of age gave informed and written consent to participate in the study. The parents, guardians or caretakers of subjects less than 18 years old gave informed and written consent for their children to participate in the study. Informed assent was obtained from children with sufficient neurodevelopmental ability.

### Clinical Data Collection

We approached all pediatric subjects who were undergoing right heart cardiac catheterization that was required for management or investigation of their underlying cardiac condition, for inclusion in the study. We excluded subjects with congenitally abnormal aortic, pulmonary, and prosthetic valves.

The heart sounds were recorded using a 3M^™^ Littmann^®^ 3200 digital stethoscope which works in conjunction with Zargis Cardioscan^™^ software (Zargis Medical Corp., Princeton, NJ, USA) to store recorded heart sounds in *. wav mono audio format. Heart sound recordings were obtained over 20 seconds with sampling frequencies of 4000 Hz. We recorded the heart sounds sequentially at the 2nd left intercostal space (2^nd^ LICS) and the cardiac apex for 20 seconds. We used the MATLAB 2010b (The MathWorks, Inc., Natick, MA, USA) programming environment for signal analysis and optimization. Heart sounds were recorded simultaneously with the direct pulmonary arterial pressure (PAP) measurements obtained during right heart catheterization in a standard manner. Other hemodynamic data including heart rate, pulmonary artery wedge pressure or left atrial pressure, right atrial pressure, oxygen consumption (VO_2_), and systemic pressure and pulmonary blood flow were collected within 5–10 minutes of the acoustic recordings.

Pulmonary arterial hypertension (PAH) was defined as a mean pulmonary arterial pressure greater than or equal to 25 mmHg with a pulmonary artery wedge pressure less than 15 mmHg according to current guidelines [19]. The heart sounds of subjects with PAH were compared with subjects undergoing cardiac catheterization but with a mean pulmonary arterial pressure less than 25 mmHg and a pulmonary artery wedge pressure less than 15 mmHg. The latter group comprised a control group with normal pulmonary arterial and wedge pressures. The clinical characteristics of these subjects have been described previously and are described in Tables 1–7 [1, 2].

**Table 1:** Pulmonary arterial hypertension: Subjects #1–11 with pulmonary arterial hypertension (mean pulmonary arterial pressure ≥ 25 mmHg).

Subject ID	Age (years)	Height (m)	Weight (kg)	BSA (m^2^)	BMI (kg/m^2^)	Gender	Diagnosis
1	0.8	0.66	6.1	0.32	14.0	M	Repaired CDH
2	0.9	0.64	5.9	0.31	14.4	F	Unrepaired CHD
3	2	0.88	11.9	0.53	15.5	M	IPAH
4	3	0.90	12.3	0.55	15.2	M	Unrepaired CHD
5	7	1.23	23	0.89	15.2	F	IPAH
6	12	1.62	62	1.66	23.6	F	Repaired CHD
7	8	1.33	33.2	1.1	18.8	M	IPAH
8	9	1.34	29.9	1.06	16.7	F	Repaired CHD
9	12	1.62	62	1.66	23.6	F	Repaired CHD
10	12	1.49	59	1.53	26.6	M	IPAH
11	15	1.30	31.7	1.06	18.8	F	IPAH
Median	8	1.3	29.9	1.06	16.7	5M:6F	
Minimum	0.8	0.64	5.9	0.31	14.0		
Maximum	15	1.62	62	1.66	26.6		

Abbreviations: BMI = Body Mass Index, BSA = Body Surface Area, CDH = Congenital Diaphragmatic Hernia, CHD = Congenital Heart Disease, F = Female, M = Male, m = meters, IPAH = Idiopathic Pulmonary Hypertension, kg = kilograms,

**Table 2:** Subjects #12–22 with normal pulmonary arterial pressures (mean pulmonary arterial pressure <25mmHg).

Subject ID	Age (years)	Height (m)	Weight (kg)	BSA (m^2^)	BMI (kg/m^2^)	Gender	Diagnosis
12	0.8	0.71	8.3	0.39	16.5	M	Unrepaired CHD
13	2	0.77	9.8	0.44	16.7	M	Repaired CHD
14	3	1.01	18.1	0.7	17.7	M	Unrepaired CHD
15	0.25	0.52	4.5	0.24	16.6	F	Repaired CHD
16	2	0.87	11.4	0.51	15.1	F	Unrepaired CHD
17	5	1.17	19	0.79	13.9	F	Post heart transplant
18	3	0.89	12.8	0.55	16.2	F	Post heart transplant
19	10	1.29	31.5	1.06	18.9	F	Post heart transplant
20	17	1.58	59	1.6	23.6	F	Repaired CHD
21	17	1.62	42	1.4	16.0	F	Repaired CHD
22	19	1.75	59	1.72	19.3	M	Post heart transplant
Median	3	1.01	18.1	0.7	16.6	4M:7F	
Minimum	0.3	0.52	4.5	0.24	13.9		
Maximum	19	1.75	59	1.72	23.6		

Abbreviations: BMI = Body Mass Index, BSA = Body Surface Area, CDH = Congenital Diaphragmatic Hernia, CHD = Congenital Heart Disease, F = Female, M = Male, m = meters, kg = kilograms.

**Table 3:** Pulmonary Vascular Hemodynamic data. Subjects #1–11 with Pulmonary arterial hypertension (mean pulmonary arterial pressure ≥25 mmHg).

Subject ID	Mean PAp (mmHg)	Systolic PAp (mmHg)	Diastolic PAp (mmHg)	Mean LAp/PAWp (mmHg)	PVRI (WUm^2^)	QPI (L/min/m^2^)
1	29	48	13	6	4.8	4.8
2	25	38	12	2	5.2	4.4
3	64	89	34	9	13.1	4.2
4	66	92	47	7	10.7	5.5
5	25	31	19	7	5.5	3.3
6	97	140	66	10	27.2	3.2
7	37	49	26	10	9.3	2.9
8	30	46	14	5	7.4	3.4
9	85	119	57	6	27.2	2.9
10	63	95	37	7	19.3	2.9
11	55	99	37	6	16.7	2.9
Median	55	89	34	7	10.7	3.3
Minimum	25	31	12	2	4.8	2.9
Maximum	97	140	66	10	27.2	5.5

Abbreviations: LAp = Left atrial pressure, L/min/m^2^ = Liters per minute per meter squared, PAp = Pulmonary arterial pressure, PAWp = pulmonary artery wedge pressure, PVRI = pulmonary vascular resistance index, QPI = Pulmonary blood flow index, WUm^2^ = Wood Units x meter squared, Note PVRI calculated from pressures at the time of QPI measurement not acoustic recording

**Table 4:** Pulmonary Vascular Hemodynamic data. Subjects #12–22 with normal pulmonary arterial pressures (mean pulmonary arterial pressure <25mmHg).

Subject ID	Mean PAp (mmHg)	Systolic PAp (mmHg)	Diastolic PAp (mmHg)	Mean LAp/PAWp (mmHg)	PVRI (WUm^2^)	QPI (L/min/m^2^)
12	20	29	17	11	2.8	3.2
13	20	32	11	8	3.1	3.9
14	15	25	10	4	0.8	14.4
15	15	25	7	6	2.0	4.4
16	24	34	15	9	4.8	3.1
17	14	27	7	7	N/A	N/A
18	20	30	12	10	2.6	3.8
19	8	11	5	5	1.3	2.3
20	17	31	9	7	2.8	3.6
21	12	22	4	5	1.6	4.5
22	14	20	8	10	1.5	2.7
Median	15	27	9	7	2	4
Minimum	8	11	4	4	0.8	2.3
Maximum	24	34	17	11	4.8	14.4

Abbreviations: LAp = Left atrial pressure, L/min/m^2^ = Liters per minute per meter squared, N/A = Not Available, PAp = Pulmonary arterial pressure, PAWp = pulmonary arterial wedge pressure, PVRI = pulmonary vascular resistance index, QPI = Pulmonary blood flow index, WUm^2^ = Wood Units x meter squared. Note PVRI calculated from pressures at the time of QPI measurement not acoustic recording

**Table 5:** Systemic Vascular Hemodynamic and Electrocardiographic data. Subjects #1–11 with pulmonary arterial hypertension (mean pulmonary arterial pressure ≥25 mmHg).

Subject ID	Mean BP (mmHg)	Systolic BP (mmHg)	Diastolic BP (mmHg)	Mean RAp (mmHg)	Heart rate (beats/min)	QRS duration (msec)	PR interval (msec)
1	59	83	41	2	130	62	66
2	68	93	47	1	130	75	91
3	48	70	34	8	99	97	98
4	70	82	56	7	115	77	92
5	70	97	50	3	66	71	88
6	93	122	73	5	107	71	71
7	96	44	67	3	75	110	106
8	62	93	42	4	65	110	88
9	88	106	71	6	90	71	71
10	78	110	56	4	70	77	71
11	68	99	53	3	80	132	110
Median	70	93	53	4	90	77	88
Minimum	48	44	34	1	65	62	66
Maximum	96	122	73	8	130	132	110

Abbreviations: BP = Systemic blood pressure, msec = milliseconds, min = minute, RAp = Right atrial pressure.

**Table 6:** Systemic Vascular Hemodynamic and Electrocardiographic data. Subjects #12–22 with normal pulmonary arterial pressures (mean pulmonary arterial pressure <25 mmHg).

Subject ID	Mean BP (mmHg)	Systolic BP (mmHg)	Diastolic BP (mmHg)	Mean RAp (mmHg)	Heart rate (beats/min)	QRS duration (msec)	PR interval (msec)
12	54	92	36	11	130	77	84
13	60	95	39	6	78	111	116
14	60	71	46	1	111	120	114
15	52	67	37	1	134	99	87
16	63	80	50	8	108	91	97
17	42	63	32	8	82	101	98
18	75	97	53	3	105	101	92
19	55	65	45	1	96	134	80
20	73	108	56	7	78	147	136
21	67	93	51	1	90	108	96
22	116	72	96	1	70	103	116
Median	60	80	46	3	96	103	97
Minimum	42	63	32	1	70	77	80
Maximum	116	108	96	11	134	147	136

Abbreviations: BP = Systemic blood pressure, msec = milliseconds, min = minute, RAp = Right atrial pressure.

**Table 7:** Comparison of clinical and hemodynamic data between subjects with pulmonary arterial hypertension (mean PAp ≥25 mmHg) and normal pulmonary arterial pressure (mean PAp <25 mmHg).

Clinical and hemodynamic variables	*p-*value
Age	0.8
Height	0.6
Weight	0.5
Body Surface Area	0.6
Body Mass Index	0.9
Systolic Pulmonary arterial pressure	<0.001**
Diastolic Pulmonary arterial pressure	<0.001**
Mean Pulmonary arterial pressure	<0.001**
Pulmonary Vascular Resistance Index	<0.001**
Pulmonary Blood Flow Index	0.9
Mean Left Atrial Pressure	0.6
Mean Right Atrial Pressure	0.8
Systolic Blood Pressure	0.2
Diastolic Blood Pressure	0.2
Mean Blood Pressure	0.1
Heart rate	0.5
QRS duration V1	0.02*
PR interval lead 2	0.07

The *p*-values from the Mann-Whitney test, where * and ** indicate p < 0.05 and p < 0.005, respectively.

#### Training Set

We recorded the heart sounds in 22 subjects from 2 sites on the chest giving a total of 44 heart sound recordings, (11 subjects with mean PAP ≥ 25 mmHg and 11 subjects with mean PAP < 25 mmHg collected from two sites: 2^nd^ LICS and apex), with a total of 1,178 heartbeats. Methods I, II, III, and IV do not require a training phase. However, Method V requires a training phase, thus, we trained the algorithm on heart sound signals collected at apex from subjects with mean PAP *≥* 25 mmHg—a total of 11 recordings.

#### Testing Set

We used all 44 heart sound recordings in Methods I, II, III, and IV. In Method V, we tested the algorithm on three datasets, heart sound signals collected on the chest at the 2^nd^ LICS from subjects with mean PAP *<* 25 mmHg, heart sound signals collected at the 2^nd^ LICS from subjects with mean PAP *≥* 25 mmHg, and heart sound signals collected at the cardiac apex from subjects with mean PAP *<* 25 mmHg—a total of 33 recordings.

#### Annotation of S1 and S2

**Fig 1:**
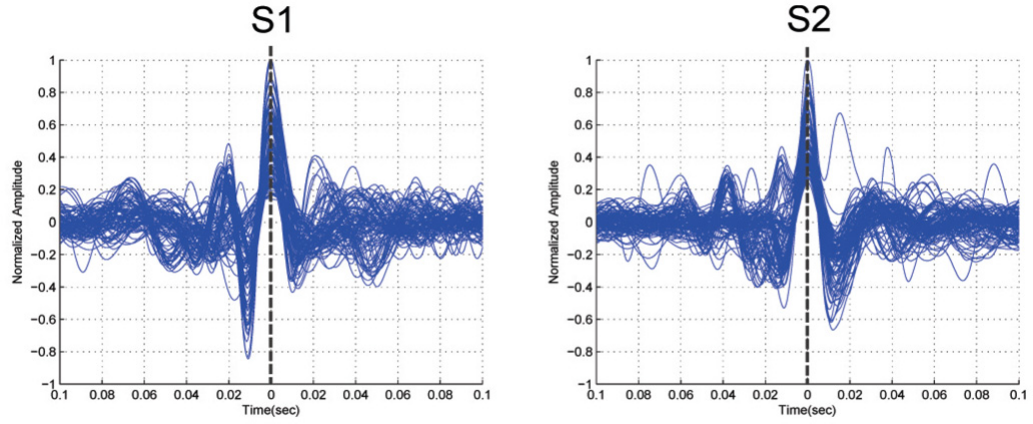
Demarcation of the 1st (S1) and 2nd (S2) heart sounds. The normalized amplitude (*y*-axis) is plotted against time in seconds (*x*-axis). Time zero second depicts the annotated peaks for S1 and S2 events.

We demarcated S1 and S2 by identifying events from the acoustic recordings that were separated by intervals compatible with the relative duration of systole and diastole. Two cardiologists identified the timing of S1 and S2 independently. They listened to acoustic recordings and marked S1 and S2 on the phonocardiographic tracing. The cardiologists’ interpretations suggested that in all subjects studied, the duration of diastole was longer than systole. Thus, the events identified as S1 and S2 occurred such that the interval between S1 and S2 was shorter than the S2 to S1 interval. Within these demarcated events, we identified the maximal (positive or negative) normalized amplitude and annotated these as reference points for S1 or S2 events, respectively. We superimposed all the events containing S1 and S2 waves with time zero at the reference point of S1 and S2 events (Fig 1).

#### Wavelet-Based S1 and S2 Detection Algorithms

We evaluated four Daubechies wavelet-based algorithms that are commonly used in the analysis of heart sound signals [20, 21]. Wavelets are closely related to filter banks. The wavelet transform (WT) of the collected heart sound signal *x*(*t*) is an integral transform defined by

$$D(a,b)=\int_{-\infty }^{\infty }x(t)\psi_{a,b}^{*}dt,$$ (1)

where *D*(*a*, *b*) is known as the wavelet *detail* coefficient at scale and location indices (*a*, *b*) of the heart sound signal *x*(*t*), ψ* denote the complex conjugate of the wavelet function *ψ*(*t*). The transform yields a *time-scale* representation similar to the *time-frequency* representation of the short-time Fourier transform (STFT). In contrast to the STFT, the WT allows a variable time and frequency resolution for different frequency bands. The set of analyzing functions—the wavelet family *ψ*_*a,b*_(*t*)—is deduced from a *mother wavelet ψ*(*t*) by:

$$\psi_{a,b}\text{(}t\text{)}=\frac{1}{\sqrt{2}}\psi \left(\frac{t-b}{a}\right),$$ (2)

**Fig 2:**
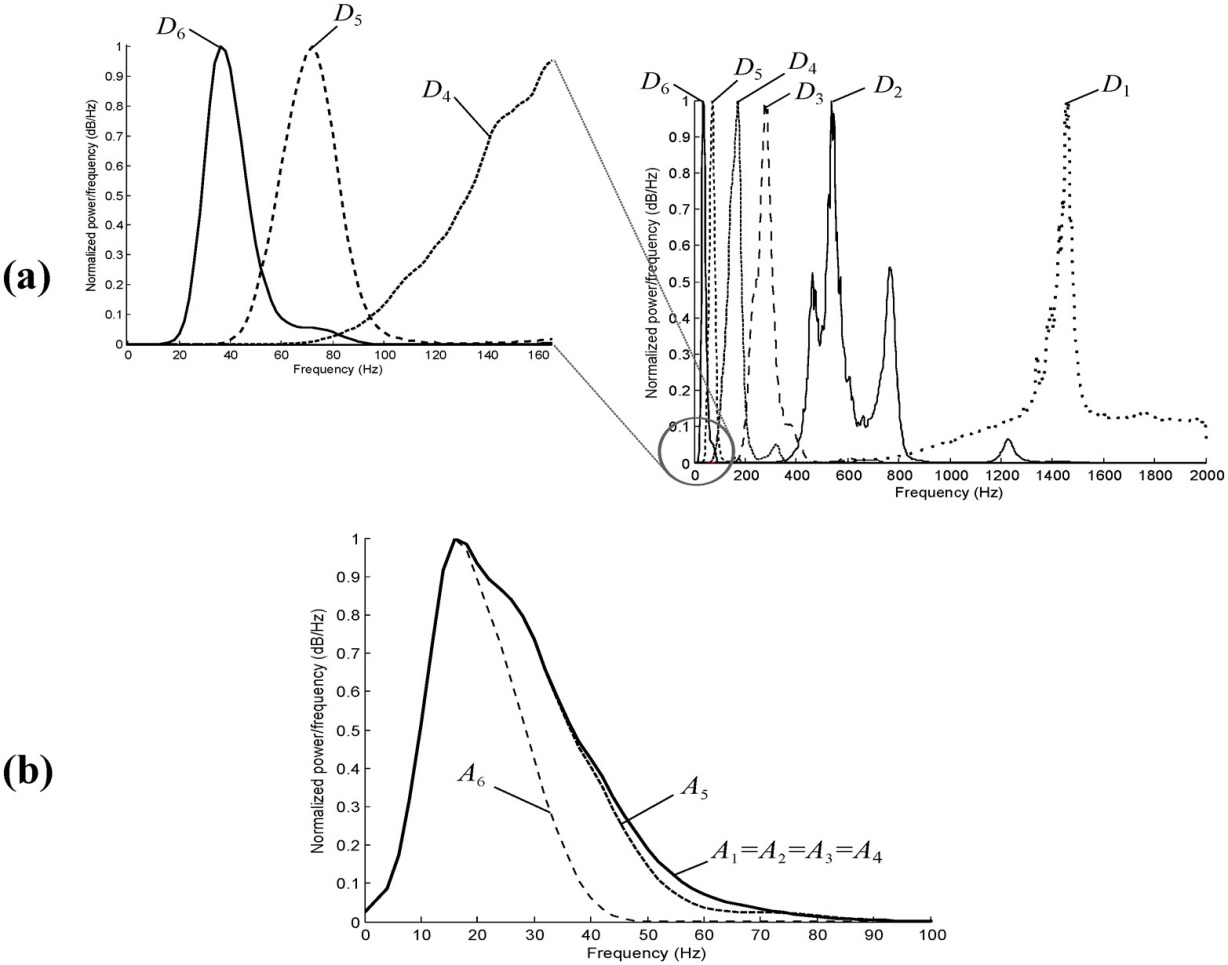
Power spectrum of ‘db6’ wavelet for details (top) and approximations (bottom) at scales *a* = 2^*j*^, *j* = 1, .., 6. Note, the sampling frequency of the heart sounds is 4000 Hz.

*a* and *b* are the *dilation* (scale) and *translation* parameters respectively. The scale parameter *a* of the WT is comparable to the frequency parameter of the STFT. The mother wavelet is a short oscillation with zero mean; note that we investigated *only* Daubechies mother wavelets. Fig 2a shows the frequency representation of the *detail* coefficients for scale *a* = 6. The orthonormal dyadic discrete wavelets are associated with *scaling functions* and their dilation equations. The scaling function is associated with the smoothing of the signal and has the same form as the wavelet, given by *ϕ*_*a*,*b*_(*t*) = 2^-*a*/2^ *ϕ*(2^-*a*^ *t*−*b*). However, the convolution of the heart sound with the scaling function produces *approximation* coefficients as follows:

$$A(a,b)=\int_{-\infty }^{\infty }x(t)\varphi_{a,b}(t)dt.$$ (3)

Fig 2b shows the frequency representation of the *approximation* coefficients for scale *a* = 6. The heart sound signal *x*(*t*) can then be represented with a combined series expansion using both the *approximation* coefficients and the *detail* coefficients, in discrete representation, as follows:

$$x(t)=\sum_{b=-\infty }^{\infty }A(a_{0},b)\varphi_{a_{0},b}(t)+\sum_{a=-\infty }^{a_{0}}\sum_{b=-\infty }^{\infty }D(a,b)\psi_{a,b}(t).$$ (4)

It is clear that the original heart sound signal is expressed as an approximation of itself, at arbitrary scale index *a*_0_, added to a succession of signal details from scales *a*_0_ down to negative infinity.

The signal *approximation* at scale *a* is defined as $x_{a}(t)=\sum_{b=-\infty }^{\infty }A(a,b)\varphi_{a,b}\left(t\right)$ while the signal details at $D_{a}(t)=\sum_{b=-\infty }^{\infty }D(a,b)\psi_{a,b}\left(t\right)$. We can write (Eq 4) as

$$x(t)=x_{a_{0}}(t)+\sum_{a=-\infty }^{a_{0}}D_{a}(t)$$ (5)

$$x_{a-1}(t)=x_{a}(t)+D_{a}(t)$$ (6)

**Fig 3:**
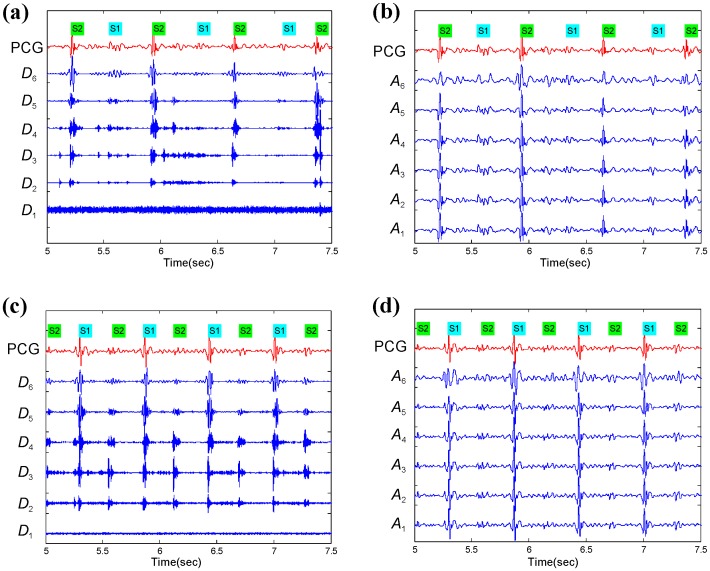
Behavior of the ‘db6’ wavelet dealing with different morphologies of S1 and S2. (a) Wavelet details for heart sounds with low S1 amplitude measured at the second intercostal space for a subject with mean PAp < 25 mmHg, (b) Wavelet approximations for the same heart sounds used in (a), (c) Wavelet details for heart sounds with low S2 amplitude measured at apex for a subject with mean PAp ≥ 25 mmHg, and (d) Wavelet approximations for the same heart sounds used in (c).

If we add the signal detail at an arbitrary scale (index *a*) to the approximation at that scale, we obtain the signal approximation at an increased resolution (i.e., at a smaller scale, index *a*−1). The time domain representations of *detail* and *approximation* coefficients are shown in Fig 3.

#### Method I: 2^nd^ order Shannon energy

**Fig 4:**
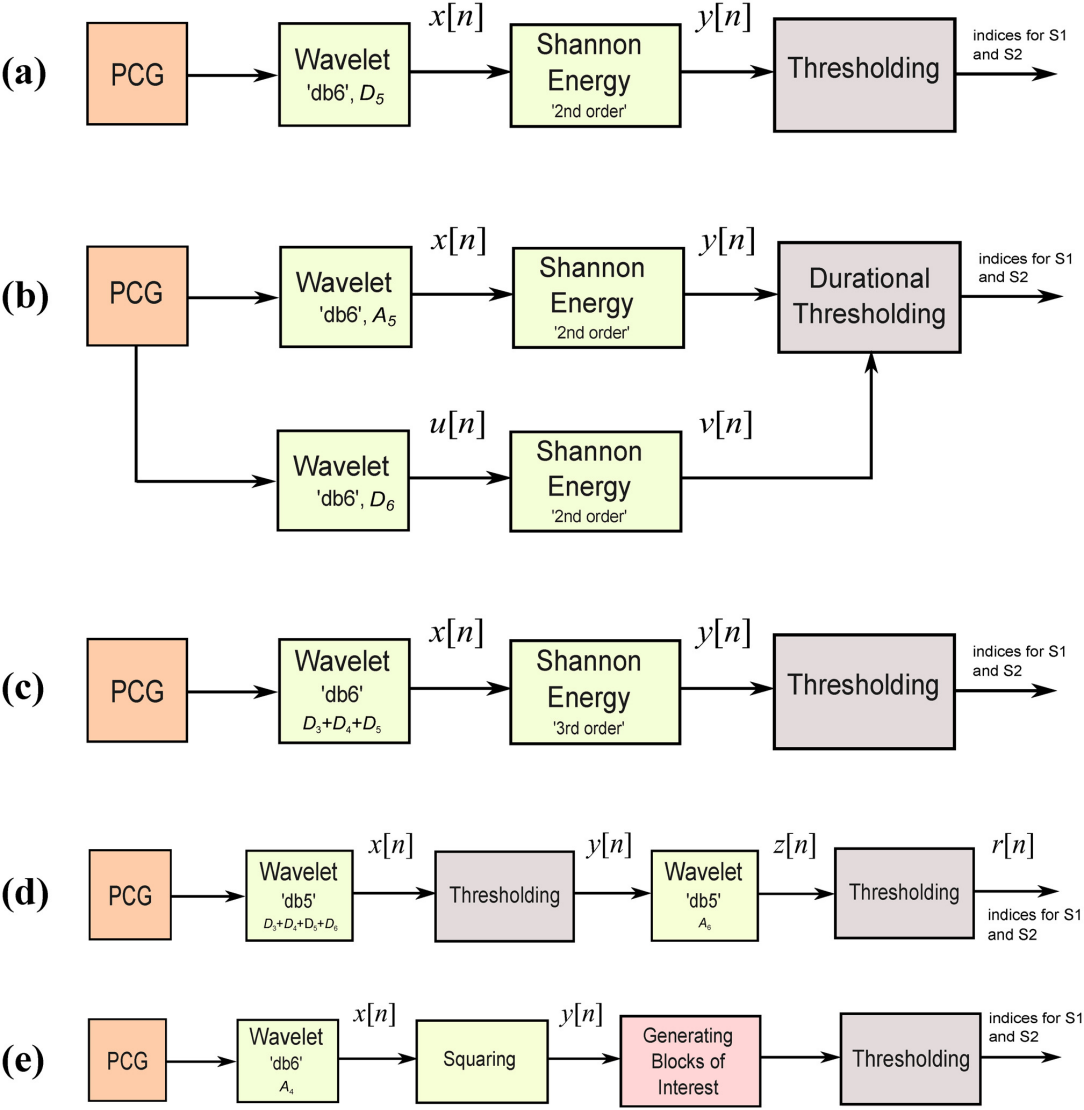
Flowcharts for five methods to detect S1 and S2 waves in heart sounds. (a) Method I, (b) Method II, (c) Method III, (d) Method IV, (e) Method V.

**Fig 5:**
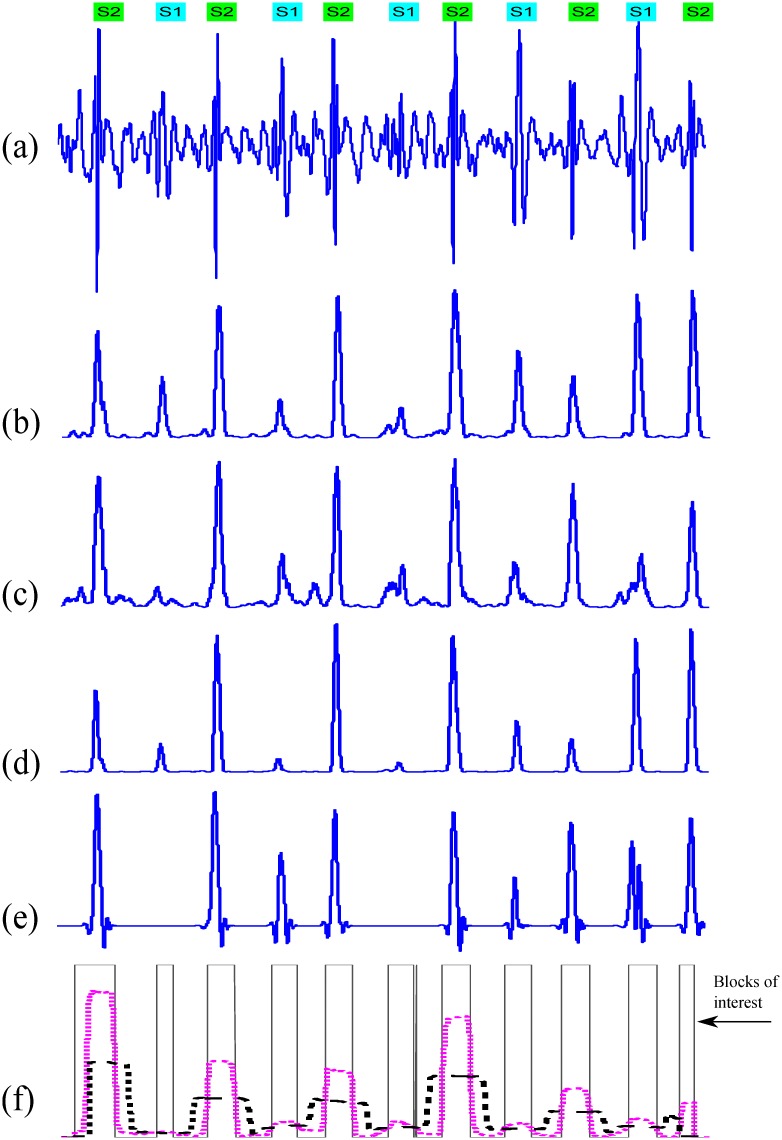
Features output. (a) Original heart sound signal from a subject with mean pulmonary arterial pressure of 20 mmHg (b) second-order Shannon energy of *D*_5_ wavelet in Method I (c) second-order Shannon energy of *D*_6_ wavelet in Method II (d) third-order Shannon energy in Method III (e) wavelet approximation *A*_6_ in Method IV (f) generating blocks of interest in Method V.

Shannon energy emphasizes medium intensity signals and attenuates low energy more than high intensity signals. It is used to detect the heart sounds and to isolate successive cardiac cycles. Liang *et al*. [12, 13] first recommended the use of Shannon energy after comparing its performance to the Shannon entropy, absolute value, and energy of heart sounds. They developed a wavelet-based algorithm that detects S1 and S2 based on the architecture shown in Fig 4a. They calculated the average Shannon energy as follows:

$$E=-\frac{1}{N}\sum_{i=1}^{N}\hat{x}{\left(i\right)}^{2}\text{log}(\hat{x}{\left(i\right)}^{2}),$$ (7)

where $\hat{x}$ is the extracted heart sound detail *D*5 of ‘db6’ wavelet normalized to the maximum absolute value of the signal $\hat{x}=\frac{D_{5}}{max(D_{5})}$, and *N* is the number of samples in 20 ms segment. Then the normalized average Shannon energy ($\hat{E}$) is computed as follows:

$$\hat{E}=\frac{E-\mu_{}}{\sigma },$$ (8)

where *μ* is the mean value of *E* and *σ* is the standard deviation of *E*. An example is shown in Fig 5, the original heart sound data (Fig 5a) and the output signal (Fig 5b) when this step is applied to detect S1 and S2 followed by a threshold $\text{THR}=\frac{1}{n}\sum_{i=1}^{n}\hat{E}(i)$.

#### Method II: 2^nd^ order Shannon energy with durational thresholds

Kumar *et al*. [8, 14] modified Method I by introducing multiple wavelet coefficients and durational threshold, as shown in Fig 4b. They extracted two wavelet features of the heart sounds: *A*_5_ and *D*_5_; and, therefore, Eq 8 is calculated twice: $\hat{E}(A_{5})$ and $\hat{E}(D_{6})$. An example of the $\hat{E}(D_{6})$ signal is shown in Fig 5c—when Method II applied to our data. An adaptive threshold THR_1_ is then introduced, based on the detail coefficients as follows

$${\text{THR}}_{1}=\hat{E}(D_{6})-\lambda \mu_{e},$$ (9)

where *μ*_*e*_ is the mean value of $\hat{E}(D_{6})$ and *λ* is a fixed value of 3. They used THR_1_ with $\hat{E}(A_{5})$ to demarcate S1 and S2. The demarcated areas were selected based on two durational thresholds: (i) THR_2_: The duration of S1 and S2 sounds is not more than 250 ms and not less than 30 ms (ii) THR_3_: The time interval between S1 and S2 is less than 50 ms. Otherwise, any event that lies outside of this interval is considered a noisy segment, and, therefore, it will be discarded from further processing.

#### Method III: 3^rd^ order Shannon energy with multiple wavelet coefficients

Wang *et al*. [22] found Method I sensitive to noise and heart murmurs that may lead to false segmentation. Therefore, they investigated different wavelet features and introduced 3rd order Shannon energy to emphasize S1 and S2 and to suppress the noise and murmurs. The flowchart of this method is shown in Fig 4c; and the average 3^rd^ order Shannon energy is calculated over 20 ms segments as

$$E=-\frac{1}{N}\sum_{i=1}^{N}\hat{x}{\left(i\right)}^{3}\text{log}(\hat{x}{\left(i\right)}^{3}),$$ (10)

The normalized average $\hat{E}$ is calculated from Eq 8, followed by a threshold THR that equals the statistical mean of $\hat{E}$. However, the $\hat{x}$ is calculated based on the summation of different wavelet coefficient features because the coefficients *D*_3_, *D*_4_, and *D*_5_ lead to a better detection accuracy. Therefore, they calculated the Shannon energy (Eq 9) with $\hat{x}=\frac{D_{3}+D_{4}+D_{5}}{\text{max}(\left|D_{3}+D_{4}+D_{5}\right|)}$, which produces the signal shown in Fig 5d—when we tested Method III on our data.

#### Method IV: Sequential Wavelet Analysis

The idea of detecting S1 and S2 using sequential wavelet analysis was introduced by Zhong and Scalzo [23]. They developed a Daubechies wavelet-based algorithm that uses the ‘db5’ wavelet, not the ‘db6’ used in Methods I, II, and III. The algorithm consists of a two-stage wavelet based on the architecture shown in Fig 4d. At the first stage, the signal equals *x* = *D*3+*D*4+*D*5+*D*6, followed by a threshold THR_1_ = 0.2, and the output signal (*y*) of this stage is a binary signal based on the equality **If** *x*[*n*]<THR_1_,*y*[*n*] = 0; **ELSE** *y*[*n*] = 1. At the second stage, the signal *Z* equals the approximation coefficients *A*6—an example is shown in Fig 5e when applied to the collected heart sound—of the *y* signal, followed by a threshold THR_2_ = 0.1, and the output signal (*r*) of this stage is another binary signal based on the equality **If** *z*[*n*]<THR_2_, *r*[*n*] = 0; **ELSE** *r*[*n*] = 1.

#### Proposed Method (Method V: Event-Related Moving Averages)

We used a novel algorithm adapted from the framework proposed for detecting QRS complexes in ECG signals, systolic wave, *a* waves, and *c*, *d*, and *e* in acceleration of photoplethysmogram signals [24–26]. The same approach was used to detect S1 and S2 events. The method consists of three main stages: pre-processing (wavelet and squaring), feature extraction (generating potential blocks using two moving averages) and classification (thresholding). The structure of the algorithm is given in Fig 4e.

#### Wavelet Choice

**Fig 6:**
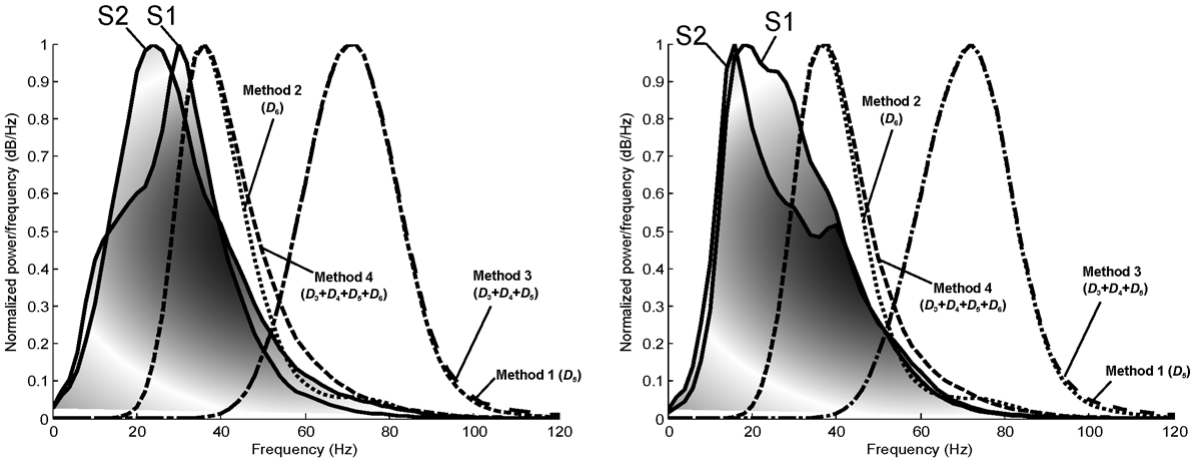
Power spectrum of S1 and S2 segments compared to the power spectrum of wavelet details used in all methods at the second intercostal space (left) and apex (right). A total of 284 heart beats used in this analysis for subjects with mean PAp > = and < 25 mmHg. Note, the sampling frequency of the heart sounds is 4000 Hz.

We selected the Daubechies wavelet base ‘db6’ because it is used most often in heart sound analysis. However, it was unclear which wavelet coefficients capture the S1 and S2 areas. Therefore, we compared the power spectrum of S1 and S2 waves with the power spectrum of the wavelet coefficients of the heart sounds (Fig 6). We found that most of the energy of S1 and S2 segments lay between 0–60 Hz. This frequency band is covered in the wavelet approximations *A*_1_, *A*_2_, *A*_3_, *A*_4_, or *A*_5_ (cf. Fig 2b).

However, it is not completely covered in the wavelet details (cf. Fig 2a). The closest wavelet detail that contains the S1 and S2 energy is *D*_6_, with the frequency range of 20–60 Hz (Fig 6). Therefore, we investigated *D*_6_ as the optimal wavelet representations for S1 and S2 events; thus, in this method, x = *D*_6_.

#### Squaring

Squaring emphasizes the large fluctuations (or magnitudes) resulting from the systolic wave, which suppress the small fluctuations arising from the diastolic wave and noise. This step results in the output

$$y\text{[}n\text{]}=x{\text{[}n\text{]}}^{2},$$ (11)

and improves the accuracy with which the systolic wave segment in heart sound signals is distinguished.

#### Generating Blocks of Interest

Blocks of interest were generated using two event-related moving averages that demarcated the S1 and S2 areas. The first moving average (MA_peak_) is used to emphasize the S1 and S2 waves area—as shown as dotted signal in Fig 5f when applied to the collected data—and is given by

$${\text{MA}}_{\text{peak}}[n]=\frac{\text{1}}{W_{\text{1}}}\text{(}y[n-\text{(}W_{\text{1}}-\text{1)}/\text{2}]+....+y[n]+....+y[n+\text{(}W_{\text{1}}-\text{1)}/\text{2}]\text{)}$$ (12)

Here, *W*_1_ represents the window size of the S1-peak or S2-peak duration. The resulting value is rounded to the nearest odd integer. The exact value for *W*_1_ of 130 ms is determined after a brute force search, which is explained in the results section. The second moving average (MA_wave_) emphasizes the beat area to be used as a threshold for the first moving average, shown as a dashed signal (Fig 5f), and is given by

$${\text{MA}}_{\text{wave}}[n]=\frac{\text{1}}{W_{\text{2}}}\text{(}y[n-\text{(}W_{\text{2}}-\text{1)}/\text{2}]+....+y[n]+....+y[n+\text{(}W_{\text{2}}-\text{1)}/\text{2}]\text{)}$$ (13)

Here, *W*_2_ represents the window size of approximately one heart sound (S1 or S2) duration. Its value is rounded to the nearest odd integer. The exact value for *W*_2_ of 270 ms is explained later in the results section.

#### Thresholding

The equation that determines the offset level (*α*) is $\beta \bar{y}$, where *β* = 0.03, is discussed later in the results section. The $\bar{y}$ is the statistical mean of the squared filtered heart sound signal. The first dynamic threshold value is calculated by shifting the MA_wave_ signal with an offset level *α*, as follows:

$${\text{THR}}_{1}={\text{MA}}_{\text{wave}}+\alpha .$$ (14)

In this stage, the blocks of interest are generated by comparing the MA_peak_ signal with THR_1_, in accordance with lines 8–15 shown in the pseudocode of algorithm V (Table 8). Many blocks of interest will be generated, some will contain the heart sound feature (S1 and S2 waves), and others will contain primarily noise. Therefore, the next step is to reject blocks that result from noise. Rejection is based on the anticipated S1/S2-peak width. In this paper, the undesired blocks were rejected using a threshold called THR_2_, which rejects the blocks that contain diastolic wave and noise. By applying the THR_2_ threshold, the accepted blocks contain S1 and S2 waves only,

$${\text{THR}}_{\text{2}}=\text{BlockSize}.$$ (15)

The threshold THR_2_ corresponds to the anticipated S1/S2 block duration. If a block is wider than or equal to THR_2_, it is classified as S1 and S2 waves. If not, it will be classified as noise. The last stage is to find the maximum absolute value within each block to detect the S1 and S2 waves; the code lines of this step are lines 17–24 in the pseudocode of algorithm V.

**Table 8:** Pseudocode of Method V. The function that detects the first heart sound (S1) and the second heart sound (S2) waves has five inputs: the heart sound signal (HS_signal_), event-related durations *W*_1_, *W*_2_, anticipated block width (BlockSize), and the offset (*β*). Daubechies 'db6' wavelet is used for filtering the signal and the wavelet detail *D*_6_ represents the heart sounds in the analysis.

Algorithm V: Detector (HS_signal_, *W*_1_, *W*_2_, BlockSize, *β*)	
1	S1 = {}, S2 = {}
2	*x* = Wavelet (HS_signal_, 'db6', *D*_6_)
3	*y* = square(*x*)
4	MA_peak_ = MA(*y*, *W*_1_)
5	MA_wave_ = MA(*y*, *W*_2_)
6	$\overset{-}{y}$ = mean(*y*)
7	*α* = $\beta \overset{-}{y}$
8	THR_1_ = MA_wave_ + *α*
9	for *n* = 1 to length(MA_peak_) do
10	if MA_peak_[*n*] > THR_1_ then
11	BlocksOfInterest[*n*] = 0.1
12	else
13	BlocksOfInterest[*n*] = 0
14	endif
15	endfor
16	Blocks = onset and offset from BlocksOfInterest
17	set THR_2_ = BlockSize
18	for *j* = 1 to number of Blocks do
19	if width(Blocks[*j*]) ≥ THR_2_ then
20	S_1,2_ = index of max. value within the block
21	else
22	ignore block
23	endif
24	endfor
25	S = Diff(S_1,2_)
26	for *k* = 1 to number of waves in S step 2 do
27	if S[*k*] < S[*k* + 1] then
28	S1 = S[*k*], S2 = S[*k* + 1]
29	else
30	S1 = S[*k* + 1], S2 = S[*k*]
31	endif
32	end for
33	return (S1, S2)

Consecutive heart sounds are shown in Fig 5f to demonstrate the use of two moving averages to generate blocks of interest. Not all of the blocks contain potential S1 and S2 waves; some blocks are caused by noise and need to be eliminated. Blocks that are smaller than the expected width for the S1 and S2 wave duration are rejected. The rejected blocks are considered to be noisy blocks, and the accepted blocks are considered to contain either S1 or S2 waves.

The last step is to determine S1 and S2 from the detected waves, which correspond to code lines 25–32 in Table 8. We differentiated the detected waves to determine S1 and S2 based on the duration among them (line 25, Table 8). To distinguish S1 from S2 blocks, another threshold is used, based on the observation that, in our subjects, diastole is longer than systole. The detected S1 and S2 waves are compared to the annotated S1 and S2 waves to determine whether they were detected correctly. The search range for the true S1 and S2 waves is fixed to *±* 5 ms for all algorithms, to ensure consistency of comparison.

## Results

We evaluated the performance of S1 and S2 wave-detection algorithms using two statistical measures: SE = 100×(TP*/*(TP + FN)) and +P = 100×(TP*/*(TP + FP)), where TP is the number of true positives (S1 and S2 waves detected as S1 and S2 waves), FN is the number of false negatives (S1/S2 waves which have not been detected), and FP is the number of false positives (non-S1/S2 waves detected as S1/S2 waves). The sensitivity SE reports the percentage of true beats that were correctly detected by the algorithm. The positive predictivity +P reports the percentage of beat detections that were true beats. The function of the S1 and S2 wave detector (cf. pseudocode of algorithm V) has five inputs: the heart sound signal (HS_signal_), event-related durations *W*_1_ and *W*_2_, anticipated block width (BlockSize), and the offset (*β*). Any change in these parameters will affect the overall performance of the proposed algorithm. These parameters are interrelated and cannot be optimized in isolation. A rigorous optimization via brute-force search, over all parameters, is conducted (cf. Table 9). As discussed in the training subsection, the data used in this training phase were heart sound signals collected at the apex from subjects with a mean PAP *≥* 25 mmHg. During the optimization phase, the window size of the first moving average (*W*_1_) varies from 20 ms to 200 ms, whereas the window size of the second moving average (*W*_2_) varies from 30 ms to 400 ms. The offset *α* was tested over the range 0–10% of the mean value of the squared filtered heart sound signal. According to our investigation (Fig 1), the duration of S1 and S2 waves is roughly 120 *±* 30 ms. The algorithm uses an optimal value of *W*_1_ (130 ms) corresponding to the duration of S1 and S2. The optimal values for the moving-average window sizes and offset are *W*_1_ = 130 ms, *W*_2_ = 270 ms, BlockSize = 60 ms, and *α* = 0.03 *z*¯ (Table 9). The S1 and S2 detection algorithm was adjusted with these optimal parameters. Then, the detector was tested on the three datasets mentioned in the training subsection without further adjustment. No adjustments were made after the detector was tested. The algorithm is fully automated and runs without the need for any human guidance.

**Table 9:** A rigorous optimization over all parameters of Method V: event-related durations *W*_1_, *W*_2_, anticipated block width (BlockSize), and the offset (*β*). All possible combinations of parameters (46,376 iterations) have been investigated and sorted in descending order according to their overall accuracy. The data used in this training phase was heart sounds measured at apex for all subjects with mean PAp ≥ 25 mmHg. The overall accuracy is the average value of SE and +P.

Iterations	*W*_1_ (sec)	*W*_2_ (sec)	BlockSize (sec)	*β* (%)	Overall Accuracy (%) (%)
1	0.13	0.27	0.06	3	80
2	0.15	0.23	0.08	1	80
3	0.16	0.24	0.08	1	80
4	0.10	0.28	0.06	4	80
5	0.13	0.24	0.07	2	80
6	0.12	0.22	0.06	2	80
7	0.14	0.22	0.06	2	80
8	0.13	0.24	0.06	2	80
9	0.11	0.24	0.06	3	80
10	0.13	0.23	0.06	2	79
11	0.13	0.28	0.06	3	79
12	0.12	0.25	0.06	3	79
13	0.11	0.27	0.07	3	79
14	0.09	0.30	0.05	5	79
15	0.09	0.31	0.02	6	79
16	0.09	0.32	0.02	6	79
17	0.12	0.26	0.06	3	79
18	0.08	0.23	0.06	4	79
19	0.09	0.29	0.04	6	79
20	0.13	0.26	0.07	2	79
.	.	.	.	.	
.	.	.	.	.	
.	.	.	.	.	
.	.	.	.	.	
46,372	0.06	0.1	0.06	0	21
46,373	0.02	0.03	0.01	0	21
46,374	0.05	0.085	0.05	0	20
46,375	0.04	0.06	0.04	0	13
46,376	0.02	0.03	0.02	0	8

We collected recordings from 22 subjects (9 males and 13 females) with a median age of 6 years (range: 3 months to 19 years). Eleven subjects had a mean PAP *<* 25 mmHg (group 1) (range 8–24 mmHg). Elven subjects had a mean PAP *≥* 25 mmHg (group 2) (range 25–97 mmHg).

**Fig 7:**
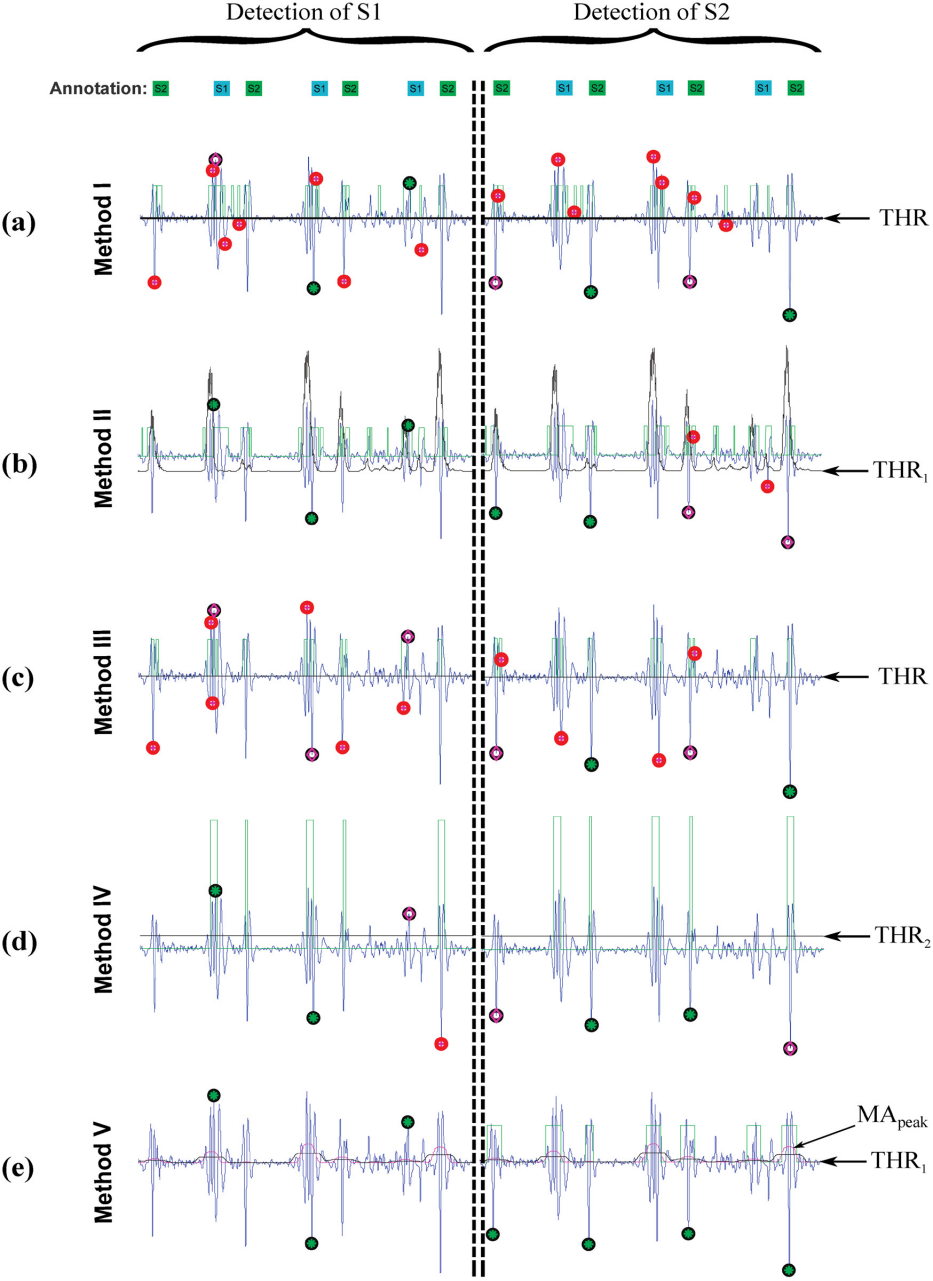
Methods performance in detecting first (S1) (left column) and second S2 (right column) heart sound waves. The black circle represents the annotated S1/S2 wave, and the green star represents the detected S1/S2 wave using each algorithm. If the black circle is empty it means a false negative, while the red circle means a false positive.

Table 10 demonstrates the performance of the described algorithms on the data collected with various mean PAP in terms of SE and +P. It also compares the advantages of each algorithm through the used wavelet coefficients, feature extraction, and thresholds. Method I introduced by Liang *et al*., which detects S1 and S2 peaks in heart sounds based on wavelet *D*_5_ feature, provides the highest detection rate after Method V. However, it is not optimal for detecting S1 and S2 peaks in heart sounds under varying conditions. Method I generates many FPs and FNs in detecting both S1 and S2 peaks after applying a fixed threshold THR, as shown in Fig 7a.

**Table 10:** Comparison of the first (S1) and second heart sound (S2) detection algorithms. To evaluate the performance of the detectors, two statistical measures were used: SE = 100×(TP/(TP+FN))and +P = 100×(TP/(TP+FP)), where TP is the number of true positives (S1/S2 detected as S1/S2), FN is the number of false negatives (S1/S2 has not been detected), and FP is the number of false positives (non-S1/S2 detected as S1/S2).

Method	WT	WT Coefficients	Feature Extraction	Threshold(s)	SE (%)	+P (%)
Method I	db6	*D*_5_	2^nd^ Shannon energy	$\text{THR}=\frac{1}{n}\sum_{i=1}^{n}\hat{E}(i)$	59	42
Method II	db6	*D*_6_	2^nd^ Shannon energy and wavelet approximation A5	${\text{THR}}_{1}=\hat{E}(D_{6})-\lambda \mu_{e}$, 250 ms > THR_2_ > 30 ms, and THR_3 =_ 50ms	19	12
Method III	db6	*D*_3_+*D*_4_ +*D*_5_	3^rd^ Shannon energy	$\text{THR}=\frac{1}{n}\sum_{i=1}^{n}\hat{E}(i)$	50	45
Method IV	db5	*D*_3_+*D*_4_+*D*_5_+*D*_6_	Wavelet approximation *A*_6_	THR_1_ = 0.2 and THR_2_ = 0.1	43	53
Method V	db6	*D*_6_	Generating blocks of interest	THR_1_ = MA_wave_+*α* and THR_2_ = BlockSize	70	68

Method II was the least accurate of the five algorithms, mainly because of the three thresholds. The first threshold (${\text{THR}}_{1}=\hat{E}(D_{6})-\lambda \mu_{e}$) shifts the feature, in most of the cases, above the *A*_5_ signal as shown in Fig 7b; thus, it provided the largest number of FPs and FNs. It seems that the use of a dynamic threshold that depends on the processed heart sound is a better idea than using a fixed threshold, as used in Method I. However, the application of the THR_1_ was unsuccessful and needs more investigation. Method III and Method IV generally incurred the same set of errors as Method I in our study, but with a smaller number of FNs, using fixed thresholds, as shown in Fig 7c and 7d. However, the proposed algorithm (Method V) scored the highest sensitivity and positive predictivity rates among the five algorithms. Two event-related moving averages, as shown in Fig 7e, may be more efficient than the THR_1_ threshold introduced in Method II and the fixed thresholds introduced in Methods I, III and IV. The proposed algorithm appears to be more robust against effects of non-stationarity, fast heart rate, and low SNR. However, the algorithm failed if S1 or S2 were low amplitude. In such cases, applying a simple level threshold is not an effective approach. The proposed Method V, however, handles varied amplitudes better than the other four algorithms and may be more amplitude-independent.

## Discussion

The purpose of our research is to develop a non-invasive screening tool for use by inexperienced clinical staff so that PAH may be detected early and result in more timely and appropriate referral to a specialist center for further evaluation by echocardiography and cardiac catheterization.

The main findings of our investigation were that a new Daubechies-based algorithm using event-related moving averages detected S1 and S2 robustly and accurately. The developed algorithm performed better than the fixed threshold methods. Moreover, it is apparent (cf. Figs 2 and 6) that the other methods did not focus on the frequency range of S1 and S2, which considerably reduced the overall performance. The sensitivity and positive predictivity of our algorithm were 69.84% and 67.87% respectively. In comparison, the sensitivity and positive predictivity of Liang’s algorithm were 58.91% and 41.82%, Kumar’s algorithm 18.88% and 11.93%, Wang’s algorithm 49.80% and 44.86%, and Zhong’s algorithm 42.68% and 52.49%. In addition, our algorithm outperformed the discussed methods up to a signal-to-noise-ratio (SNR) of 10 dB.

Our main objective was to evaluate the robustness of the algorithms against the low SNR and high heart rates of children with a mean PAP *≥* 25 mmHg. The heart sounds analysis was difficult because heart sound amplitudes varied with time, and simple level thresholds did not optimally detect S1 and S2 waves. This reduced the algorithm detection performance. Also, heart sounds may be distorted with low and high frequencies because of breathing, muscle movements, and ambient noise; however, choosing the most appropriate wavelet coefficients may minimize these confounding effects.

All of the algorithms failed to some degree. Possible reasons for this included false-negative detection of the heart sounds because of tachycardia or the fixed search range of 5 milliseconds. Alternatively there may have been false-positive detection of the heart sounds because of a decrease in amplitude (S1 has a lower amplitude than S2 or vice versa) or a low SNR.

**Fig 8:**
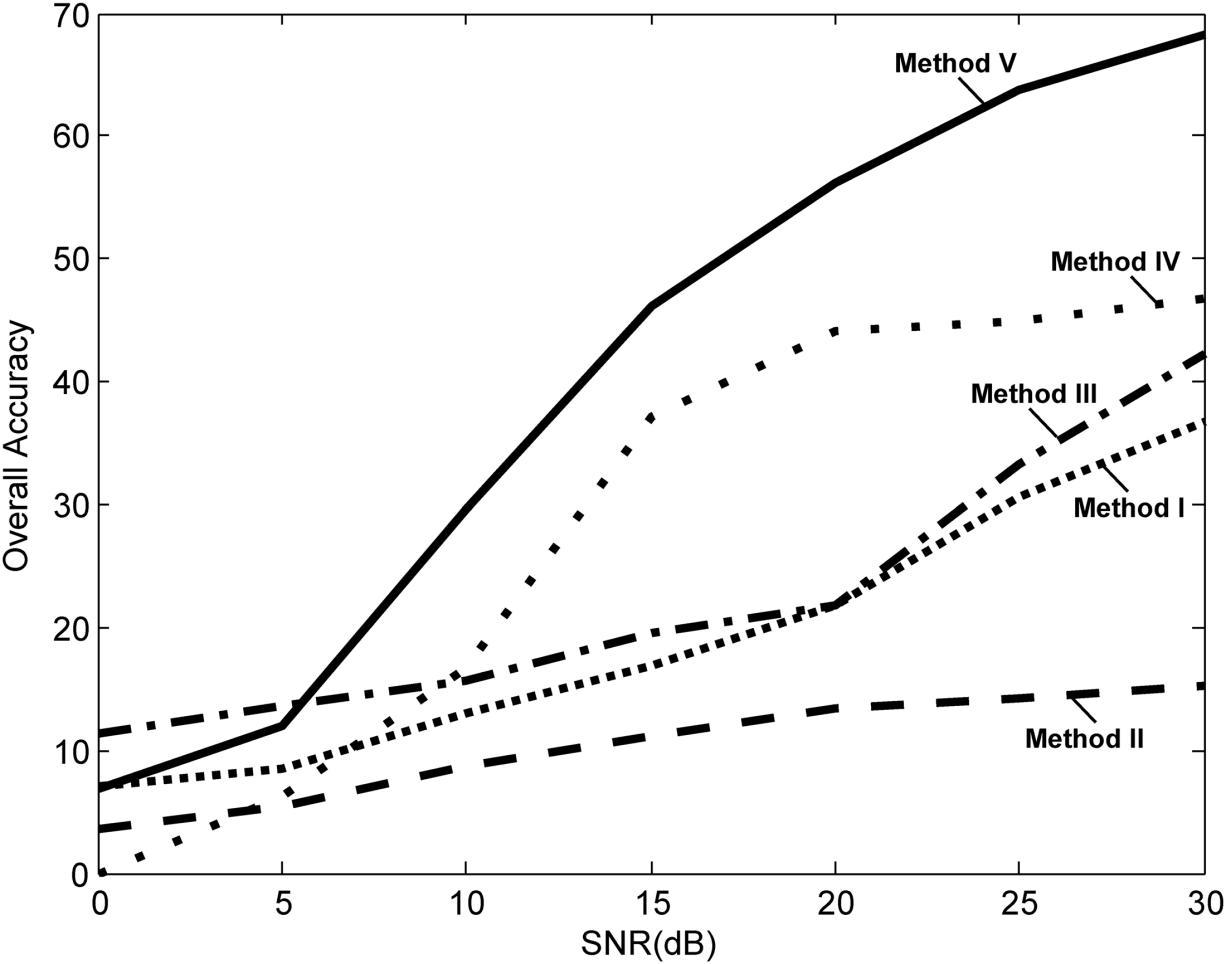
Performance at different signal-to-noise ratio (SNR) levels. It is clear that the overall accuracy of Method V increases when the SNR increases compared to the other methods.

The robustness of the five algorithms was tested against additive white Gaussian noise contamination. All heart sounds were tested against varying signal-to-noise ratios. The white noise was added above the noise collected during the heart sound measurement. Fig 8 shows the performance of the five algorithms at varying noise levels. As expected, the performance of the algorithms degraded with a decrease in signal-to-noise ratio. However, the study showed that Method II is very sensitive to noise while the proposed algorithm (Method V) outperformed the other methods up to SNR of 10 dB. The accuracy of the reported algorithms, when applied to our clinical data, is lower than has been reported in the literature. There are many factors that might account for this including the sensor, sampling frequency, and algorithm implementation. For example Kumar et al. used a Meditron stethoscope, which has a good signal to noise ratio and an extended frequency range digitized using a 16-bit analogue to digital converter at 44.1 kHz [8, 14]. Liang et al. used a customized electronic stethoscope with 16-bit accuracy using 11025 Hz sampling frequency [7, 12, 13]. Perhaps their sensors were more robust to noise compared to ours, and, therefore, the S1 and S2 waves were clearer. In addition, our stethoscope sensor has a lower sampling frequency of 4000 Hz. One of the main reasons behind the relatively low accuracy may be the implementation of the discussed algorithms. The last step of each algorithm, which determines if the detected peak could be considered as an S1 or S2 wave has not undergone robust discussion in the literature. Based on the training and testing phases (cf. 9 and 10), the proposed algorithm performed better than the other algorithms at detecting S1 and S2 peaks in all subjects with mean PAP ≥ 25 mmHg and mean PAP < 25 mmHg at both the cardiac apex and 2^nd^ LICS.

All of the algorithms we evaluated do not impose an extensive computational overhead while avoiding the manual segmentation and patient-specific modifications that are often required in biosignal analysis.

As far as we are aware the comparison of different algorithms and their performance on a single data set has not been reported.

Although our long-term goal is to non-invasively detect PAH this was not the main focus of the work described in the current manuscript. In this paper we attempted to evaluate the automatic detection of S1 and S2 in subjects with and without PAH. Our next step would be to use the heart sounds in particular the characteristics of S2 to diagnose PAH in a similar manner to clinicians who determine the presence of PAH by the behavior of the 2^nd^ heart sound. However, an open question for future studies is to explore the minimum number of accurately identified S1 and S2 events for automatic detection of PAH from the acoustic behavior of the heart sounds.

## Conclusion

A Daubechies-based algorithm using event-related moving averages detected S1 and S2 with robustness and accuracy. The developed algorithm performed better than the fixed threshold methods. An algorithm to detect S1 and S2 waves in heart sounds measured from children with and without pulmonary arterial hypertension has not been addressed in the literature. We have developed a robust algorithm for detecting S1 and S2 peaks in the heart sounds of children with low amplitude, non-stationary effects, and high heart rates. The algorithm was evaluated using 44 records, containing 1,178 heartbeats, with an overall sensitivity of 69.84% and positive predictivity of 67.87%. Based on our spectral analysis, we recommend the use of wavelet ‘*D*_6_’ detail for detecting S1 and S2 waves because it captured most of the energy contained within S1 and S2.
